# COVID-19 Surveillance of Healthcare Workers during the SARS-CoV-2 Vaccination Period: An Updated Protocol Suggestion

**DOI:** 10.6061/clinics/2021/e3096

**Published:** 2021-06-23

**Authors:** Marcos Roberto Tovani-Palone, Muhammad Farrukh Siddiqui

**Affiliations:** I Faculdade de Medicina de Ribeirao Preto, Universidade de Sao Paulo, Ribeirao Preto, SP, BR; II Liaquat National Hospital and Medical College, Karachi, Pakistan

The coronavirus disease (COVID-19) pandemic has resulted in several negative impacts globally, especially for health systems. Both the assiduity of the healthcare professionals and provision of continuous care by the teams involved must be regarded with great importance (1,2), given the high demand for outpatient and hospital services for patients with the disease (3). It is also the responsibility of the health services to manage the professionals infected by severe acute respiratory syndrome coronavirus 2 (SARS-CoV-2).

Although SARS-CoV-2 mass vaccination has already been successful in some countries, its full implementation has been a major challenge for different parts of the world (4). In health, the main objective of vaccination and surveillance is to protect healthcare workers against SARS-CoV-2 infection, to avoid absence from work because of the disease, and to prevent the occurrence of outbreaks within services. The preservation of health (relating to COVID-19) of both professionals and patients who have sought care and/or have been hospitalized for various reasons (other than COVID-19) is also relevant. In the latter case, the concern should be focused mainly on large hospitals that include both service wards for patients with COVID-19 and others for patients with various diseases (other than COVID-19).

However, there are still uncertainties regarding the cure criteria for the disease (5), which has hindered the effectiveness of COVID-19 surveillance services aimed at healthcare workers worldwide. Moreover, there is a lack of consolidated knowledge on the adverse reactions associated with different SARS-CoV-2 vaccines used in hundreds of countries. Considering that healthcare professionals who have direct contacts with patients with COVID-19 are usually part of the initial target of SARS-CoV-2 vaccination campaigns, we prepared a protocol for the surveillance of this occupational group in cases of COVID-19 suspicion and/or confirmation during the vaccination period.

## Protocol description

First, when implementing SARS-CoV-2 vaccination for healthcare workers in health services that serve patients with COVID-19, some specific actions are required. Ideally, the real-time polymerase chain reaction test should be performed for all COVID-19 suspected cases before vaccination, as it is possible that the healthcare professional may have been previously infected with SARS-CoV-2 and appears asymptomatic. In the absence of prior testing, some professionals who receive the vaccination may be asymptomatic infected patients and may subsequently develop clinical symptoms of the disease, resulting in ineffective vaccination, waste of inputs, and costs. Table 1 describes additional actions related to the surveillance of healthcare workers during the vaccination period.

## Definition of a suspicious case

A suspected case should include a healthcare worker in health services (that serve patients with COVID-19) who had contact with suspected COVID-19 or positive cases during work, without the use of specific personal protective equipment (specifically N95/PFF2 mask, goggles, and disposable lab coat), or who had contact with suspected or positive cases of the disease outside the health services without the use of an N95/PFF2 mask. In both cases, regardless of the presence or absence of symptoms, COVID-19 should be suspected. Given the greater transmissibility of the new variants of the virus (6,7), the use of masks with greater protection is essential for healthcare workers (8).

## Conduct in specific situations

If symptoms persist in those who are tested negative, retesting is recommended. Furthermore, when a healthcare worker is sampled to investigate the presence or absence of the disease, the healthcare worker and those who live in the same household must remain in home isolation for a period, in line with the recommendations of their respective national health surveillance institutions.

Finally, we emphasized that it is also important to use masks that provide greater protection (N95/PFF2) for all professionals in all departments of health services that serve patients with COVID-19, including those in administrative roles. This is essential. Inevitable contacts between professionals who provide direct care to infected patients with others are not uncommon. Hence, there is a greater risk of extensive circulation of the virus within the health services and greater risks of cross-infection.

## Figures and Tables

**Table 1:** Recommendations for the surveillance of symptomatic professionals in health services (that serve patients with COVID-19) during the SARS-CoV-2 vaccination period.

During the vaccination period, if:	Actions
• Mild symptoms	- Monitor cases until total remission of symptoms, together with home isolation.
• Moderate to severe symptoms	- RT- PCR*.

* Real-time polymerase chain reaction.
